# Changes in temporal sensory profile, liking, satiety, and postconsumption attributes of yogurt with natural sweeteners

**DOI:** 10.1111/1750-3841.16224

**Published:** 2022-06-16

**Authors:** Diksha Chadha, Nazimah Hamid, Kevin Kantono, Manon Marsan

**Affiliations:** ^1^ Department of Food Science, Faculty of Health and Environment Sciences Auckland University of Technology Auckland New Zealand; ^2^ Agrocampus Ouest Rennex Cedex France

**Keywords:** hunger, monk fruit, multiple‐intake TDS, purchase intent, satisfaction, xylitol

## Abstract

**Abstract:**

Sweetened yogurts can contain between 10 and 13% added sugar. However, studies have shown that sugar reduction or replacement can influence yogurt quality. The main objective of this research was to investigate the effects of yogurt with added natural sweeteners on temporal sensory profile, liking, satiety and postconsumption measures. Yogurt samples were prepared with iso‐sweet concentrations of sucrose (9 g/100 g of plain yogurt) using xylitol (10 g/100 g), stevia (0.15 g/100 g), and monk fruit (0.15 g/100 g). Fifty panelists evaluated the temporal sensory profile of these yogurts using multiple‐intake temporal dominance of sensations (TDS), and overall liking for each intake. In addition, satiety (hunger, thirst, and fullness) and other postconsumption attributes (healthiness, satisfaction, and purchase intent) were determined. The temporal profile of yogurt sweetened with xylitol was similar to yogurt sweetened with sucrose without any onset of negative sensory characteristics at any point in intake. Yogurt sweetened with stevia had a high dominance duration for astringency. Moreover, yogurt sweetened with monk fruit showed increased dominance of attributes *bitter* and *astringent* from the first to third intake. In terms of liking, yogurt containing xylitol was scored the highest followed by stevia and monkfruit. *Sweet* was a positive temporal driver of liking in yogurt sweetened with monk fruit. However, *mouthcoating, sweet*, and *sour* decreased liking in yogurt sweetened with sucrose, xylitol, and stevia respectively. In terms of perceived healthiness, satisfaction and purchase intent, yogurt sweetened with sucrose scored the highest followed by xylitol. Consumption of yogurt sweetened with xylitol, stevia, or monk fruit significantly decreased hunger compared to yogurt sweetened with sucrose.

**Practical Application:**

The current findings will play an important role for the dairy industry in understanding how sugar replacement with natural sweeteners in yogurt can influence its sensory perception and postconsumption behavior.

## 1 INTRODUCTION

Yogurt is widely consumed because of its numerous health benefits, affordability, and their availability in various flavors. Consumers are increasingly aware about the health concerns associated with increased consumption of sugar. As reduction of sugar would be beneficial to health, there has been increasing research investigating how the use of no or low sugar alternatives in yogurt influenced eating quality. Gille et al. (2012) reported that 51% of Swiss panelists above 50 years of age found that flavoured yogurts available in the local market were too sweet. Moore et al. (2018) further reported on the total sugar content of flavoured yogurt (12%), children's yogurt (10.8%), fruit yogurt (11.9%), and organic yogurt (13.1%) products in UK supermarkets. These values exceed the recommendation of no more than 10% of energy from sugar following guidelines of the World Health Organization of (Johnson et al., 2009; Mathers, 2008), and accounted for more than 45% of the total energy intake (Moore et al., 2018).

The use of sugar substitutes like nutritive and intensive sweeteners is the most common technique of sugar reduction. However, because of the various functions of sugar, removing sugar from food products not only influences sweetness, but can also affect the overall functionality, flavour perception, texture, and overall liking of food (Hutchings et al., 2019; Markey et al., 2015). Intensity and persistence of sweetness, and aftertaste of sweeteners can vary depending on the type of food products (Choi & Chung, 2015; de Oliveira Rocha & Bolini, 2015; Heikel et al., 2012), sweetener concentration (Cardello et al., 1999), and tasting conditions, such as product consumed at different temperatures or with varying fat content (Paixão et al., 2014). Recently, there has been an increasing trend among certain consumer segments to avoid artificially produced sweeteners such as sucralose and aspartame. As a result, there is a drive toward the use of natural high‐potency sweeteners like stevia in yogurt (Costa et al., 2019; Pereira et al., 2021), protein beverage (Harwood & Drake, 2021; Parker et al., 2018), and chocolate ice‐cream (de Medeiros et al., 2020); as well as monk fruit in chocolate milk (Li et al., 2015), protein beverage (Harwood & Drake, 2021; Parker et al., 2018), and yogurt (Ban et al., 2020). The use of xylitol as a natural sweetener is somewhat limited, and has only been reported in studies on yogurt (Costa et al., 2019; da Costa et al., 2020) and cookies (Winkelhausen et al., 2007). The present study focused on the use of “natural sweeteners” like xylitol, stevia, and monk fruit in yogurt as a sugar substitute.

Changes in sensory attributes over time can provide a better understanding of the dynamic characteristics of products reformulated using natural sweeteners. Temporal dominance of sensations (TDS) can characterise the dominant sensory sensations perceived by panelists during the consumption of a product over a specified time (Meyners, 2011). This method has been successfully used to characterize the dynamic sensory characteristics of dairy products such as chocolate dairy dessert (Morais et al., 2014), yogurt (Greis et al., 2020; Lesme et al., 2020; Oliveira et al., 2021; Pereira et al., 2021), Prato cheese (Rodrigues et al., 2021), and chocolates (Rodrigues et al., 2016). Moreover, the evaluation of products using multiple intake TDS is important as food products are consumed in repeated bites, which can significantly change how they are perceived due to sensory adaptation. Lesme et al. (2020) found that the overall flavour perception of yogurts varied significantly with three intakes, which in turn impacted the dominance of sweetness and sourness. Studies on sweeteners have mainly used time‐intensity (TI) analysis to evaluate changes in sweetness intensity in ice cream sweetened with sucralose or stevia (de Medeiros et al., 2020), chocolate dairy dessert sweetened with a range of sweeteners (sucralose, neotame, stevia, and aspartame) (Morais et al., 2014), and yogurts sweetened with stevia or thaumatin (Pereira et al., 2021). However, the focus on a single attribute during the evaluation period can result in the loss of significant information about changes in other sensory attributes. Hence, temporal methods like TDS and TCATA (Temporal Check‐All‐That‐Apply) are advantageous in evaluating products formulated with sweeteners as a more complete temporal profile of important sensory attributes can be obtained within a single session with the panelists.

Artificial sweeteners are metabolized differently due to their different properties. As a result, there are conflicting findings reported regarding their effects on body weight control, glucose homeostasis, and underlying biological mechanisms (Pang et al., 2020). Previous studies have demonstrated the effect of sweeteners on energy intake, postprandial glucose response, insulin, and blood glucose levels (Anton et al., 2010; Farhat et al., 2019; Tey et al., 2017). Anton et al. (2010) found that consumption of stevia before lunch and dinner significantly reduced the postprandial glucose levels compared to consumption of sucrose. In addition, the postprandial insulin levels are also significantly reduced compared to consumption of both sucrose and aspartame (Anton et al., 2010). Farhat et al. (2019) further found that consumption of stevia before an ad libitum pizza lunch lowered the appetite sensation without any further increase in the postprandial glucose levels. It has been shown that beverages formulated with nonnutritive sweeteners (aspartame, stevia, and monk fruit) that were consumed 1 h before lunch did not have any significant effect on the total daily energy intake, postprandial glucose, and insulin levels compared to sucrose sweetened beverage (Tey et al., 2017). None of these studies to our knowledge have investigated how a food product formulated with sweeteners influenced the hunger, thirst, and fullness status of consumers. Only two studies have investigated the effect of nutritional supplement consumption on hunger and thirst (Regan et al., 2021; Thomas et al., 2018). Thomas et al. (2018) found that oral nutritional supplements varying in energy density and volume over 10 sips marginally reduced the hunger status and significantly increased the thirst status in older adults aged between 60 to 75 years. Regan et al. (2021) further reported that consumption of a vanilla flavoured ready‐to‐drink beverage style oral nutritional supplement over five sips resulted in significantly lower appetite in adults above 75 years old than the 65–74 years old. Hence the evaluation of self‐reported or subjective hunger, thirst, and fullness status of participants before and after the consumption of yogurts formulated with different sweeteners was carried out in this study.

Replacement of sugar in food products can be challenging to food technologists during product development as it can influence sensory perception, product quality, and consumer acceptability. Therefore, the main objective of this research was to investigate how sugar reduction in yogurt can influence temporal sensory perception and liking. Moreover, to gain a better understanding of consumer food choices, the present research also investigated the effects of sweetener type on healthiness, satisfaction, and purchase intent after the consumption of yogurt. In this study, it was hypothesized that the use of different sweeteners in yogurts would influence the temporal changes in dominant sensory attributes over time. These changes may further impact the postconsumption attributes and purchase intent of yogurts containing different sweeteners.

## 2 MATERIALS AND METHODS

### 2.1 Ethics statement

Ethical approval (20/73) for the present research was obtained from the Auckland University of Technology Ethics Committee (AUTEC). A written consent was provided by all the panelists before the commencement of data collection.

### 2.2 Materials

Yoplait Greek style natural yogurt (General Mills, Golden Valley, MN, USA) bought from Countdown, Auckland, New Zealand was used in this study. The sugar substitutes used were xylitol (NOW Foods, Real food, pure granulated xylitol), stevia (NOW, Better Stevia, Certified organic stevia leaf extract), and monk fruit (NuNaturals, pure monk fruit (*Luo Han Guo*) extract) were purchased from iHerb, Moreno Valley, CA, USA. Sucrose (Chelsea white sugar) was obtained from a local supermarket in Auckland, New Zealand.

### 2.3 Panelists

For TDS evaluation, 50 panelists (17 men and 33 women), aged between 21 and 50 years old participated in this study. Panelists were recruited through poster advertisements on social media networks (Facebook and Instagram) and around the campus. They were rewarded with supermarket voucher for their participation. Panelists who followed vegetarian, vegan, or kosher diet, had any medical conditions associated with food or any other food allergies were excluded from this study. The data collection was carried over a 2‐month period (September and October 2019). Both training and data collection were performed between the hours of 9:00 a.m. to 1:00 p.m. on the weekdays in the sensory laboratory located at the Food Science Department, Auckland University of Technology.

### 2.4 Yogurt samples

#### 2.4.1 Iso‐sweet determination

The iso sweet concentration of the natural sweeteners in relation to sucrose was conducted according to the “Difference from Reference method” (Di Monaco et al., 2014). This method was used to determine the concentrations of natural sweeteners that are equivalent to the sweetness intensity of 9 g of sucrose in 100 g of plain yogurt (Saint‐Eve et al., 2016). A total of 15 panelists aged between 21 and 50 years of age participated in this test. In reference to previous literature (Ban et al., 2020; Costa et al., 2019; Pereira et al., 2021), two concentrations of xylitol (9 g and 10 g/100 g), stevia (0.15 g and 0.20 g/100 g), and monk fruit (0.10 g and 0.15 g/100 g) were prepared and evaluated using the sip and spit method (Miele et al., 2019). Each sample was served at room temperature in plastic cups coded with three‐digit random numbers.

Panelists were asked to consume the reference sample first (sucrose 9 g/100 g) and then the other samples. For each sample, panelists indicated whether the sweetness intensity of yogurts containing alternative natural sweeteners was less than, greater than or equal to the reference yogurt sweetened with 9% sucrose. A 10 cm linear scale anchored with 0 (far less sweet than reference), 5 (sweet as reference), and 10 (far much sweeter than reference) was used. Panelists were requested to cleanse their palate by drinking water or eating crackers in between samples and waited at least 30 s before the next evaluation. Using this method, the plain yogurt samples sweetened individually with xylitol (10 g/100 g), stevia (0.15 g/100 g), and monk fruit (0.15 g/100 g) were determined to have equivalent iso‐sweetness to sucrose (9 g/100 g). These were the concentrations of sweeteners used in this study.

#### 2.4.2 Preparation of samples

The present study used an unsweetened Yoplait Greek‐style natural yogurt obtained from a local supermarket in Auckland, New Zealand. The sweeteners were added to the yogurt a day before sensory testing. Plain yogurt was stirred using a glass rod instantly after the addition of sucrose or sweeteners until they dissolved completely (Ribeiro et al., 2020). Samples were then packaged in polystyrene cups and stored in commercial‐grade fridge (Fisher and Paykel, East Tamaki, New Zealand) at 4°C for 24 h to maintain consistency. After 24 h, 50 g of each sample was served in plastic cups coded with three‐digit random codes, with presentation counterbalanced and randomized across panelists (Bower & Baxter, 2000).

### 2.5 Multiple‐intake temporal dominance of sensations

The present study used the multiple intake temporal dominance of sensations (TDS) method to determine temporal changes in the sensory sensations as described by Jager et al. (2014). Intensity scales were substituted with buttons that corresponded to the different sensory attributes. TDS data was successively binary coded across time, with 0 corresponding to unselected attribute and 1 corresponding to selected attribute. To adhere to the concept of dominance, if one button corresponding to single sensory attribute was selected, the other buttons automatically became deselected. The sensory attributes that were evaluated in this study over a period of 45 s were sweet, sour, bitter, creamy, fruity, mouthcoating, licorice, and astringent. Definitions and references for these sensory attributes were determined by the panelists during training (Table 1). TDS‐related best‐practices were implemented, that is, short product evaluation time (45 s) along with the diverse range and consistent order of sensory attributes (Pineau et al., 2012).

**TABLE 1:** Definition and references of the attributes for TDS evaluation of yogurts

Attribute	Definition	Reference
Sweet	Sensation associated with the presence of sugars	2% Sucrose solution
Sour	Sensation associated with the taste of fermented dairy products or citrus fruits	0.08% citric acid solution
Bitter	Sensation associated with bitter taste	0.05% caffeine solution
Creamy	Sensation associated with full, soft, and smooth texture	Milk with 20% added milk cream
Fruity	Sensation associated with sweet, floral, and aromatic blend	Ripe fruits like peaches and apricots
Mouthcoating	Sensation associated with adhesion of the product to the palate and teeth	Sour cream
Licorice	Sensation associated with alcohol solution	One‐fourth tsp aniseed
Astringent	Sensation associated with a dry and rough feeling on the tongue and oral cavity	Tannic acid (3.0 g/L) in water

#### 2.5.1 Panel training

Panel training was carried out over two different sessions. In the first session, panelists were showed a demonstration video of how TDS will be carried out. Panelists then identified, defined, and familiarized themselves with the sensory attributes that described the sweetened yogurts. The concept of “dominant attribute” was explained to the panelists and described as an attribute associated with the sensory sensation that catches their attention at any given time. Panelists were informed that dominance might change if they perceive a new sensation (Labbe et al., 2009; Pineau et al., 2009). In the second session, panelists were trained to carry out the multiple intake TDS procedure. A dummy TDS trial was carried out in which panelists were asked to consume a plain yogurt sample sweetened with sucrose and evaluated the dominant sensory sensations over 45 s over three intakes. This allowed the panelists to familiarise themselves with the TDS methodology and computer interface. During the whole training session, the panel leader actively reinforced panelists’ understanding of the procedure and assisted them wherever necessary.

#### 2.5.2 Yogurt evaluation using multiple intake TDS

Panelists clicked the “START” button on the left‐hand side of the computer screen at the start of the experiment. On‐screen instructions were provided to minimize the variation in the eating behavior of panelists. The instructions given were: “Take a spoonful of sample and keep it in your mouth for 20 s,” and after 20 s “Please swallow the sample.” After swallowing, panelists clicked on the dominant sensory attribute at a given time, with the instruction “Keep clicking on the dominant attribute.” Panelists selected a new dominant attribute once a change in dominant sensation was detected. Panelists were free to choose the same attribute multiple times or to not select any attribute as dominant. TDS evaluation of each sample was carried for 45 s out over three different intakes. Similar steps were followed to evaluate the second and third intakes of the yogurts.

### 2.6 Experimental design

First of all, panelists indicated their respective baseline measurements for hunger, thirst, and fullness using a 100 mm line scale ranging from “not at all” to “extremely” (Flint et al., 2000). Then each sample was evaluated by TDS for 45 s over three different intakes as described in Section 2.5.2. The entire TDS evaluation procedure was performed in two different sessions, each of them typically lasting for 20 min with a 30 min break in between sessions. Yogurts formulated with sucrose and xylitol were evaluated in the first session. Yogurts formulated with stevia and monk fruit were evaluated in the second session.

After TDS evaluation, panelists rated overall liking for the sample using a nine‐point hedonic scale ranging from “extremely dislike” to “extremely like” for each intake. This method of measuring liking after each intake is known as alternated temporal drivers of liking (A‐TDL) (Thomas et al., 2016). It provides a better understanding of consumer liking and permits the correlation of the hedonic dynamic profile of each intake with the individual TDS profile.

After the TDS evaluation and rating of overall liking in the third intake, panelists evaluated hunger, thirst, and fullness (Flint et al., 2000). Finally, panelists rated the perceived healthiness, purchase intent, and satisfaction of the yogurt samples using a 100 mm line scale ranging from “not at all” to “extremely.”

**FIGURE 1:**
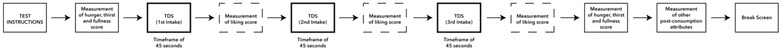
Description of evaluation process of each yogurt without any limited timeframe for each screen except for the TDS screen

The entire evaluation procedure has been summarised in Figure 1. The panelists were given a compulsory 30‐s break in between tasting the different yogurt samples. Water and water crackers (Countdown, Auckland, New Zealand) were provided for palate cleansing. This time interval between each sample was selected after preliminary trials to ensure that no carryover of flavour occurred before the next sample was tasted. The entire data collection was performed using the Fizz Acquisition software (Version 2.46b, Biosystemes, Couternon, France).

### 2.7 Data analysis

All univariate and multivariate analysis in the present study were performed using the XLSTAT Sensory software (version 2020.3.1) (Addinsoft, Long Island City, NY, USA).

#### 2.7.1 TDS curves

The dominance rating of each attribute by each panelist was plotted as a function of time using the in‐built spline‐based smoothing algorithm (Lenfant et al., 2009) using the Fizz Acquisition software (Version 2.46b, Biosystemes, Couternon, France). The chance level (*P*_0_) was defined as the dominance rate that an attribute can obtain by chance considering all the attributes evaluated. Significance level (*P_s_*) was defined as minimum value of dominance rate that an attribute has to attain to be significantly higher than *P*_0_ (Pineau et al., 2009). TDS time was presented as standardized time (Vidal et al., 2016), and data were transformed to percentages (0–100%).

#### 2.7.2 Canonical variate analysis

Canonical variate analysis (CVA) was carried out to inspect the differences in duration of the attribute's dominance in the product and within each intake of the product. Furthermore, Hotelling–Lawley multivariate analysis of variance (MANOVA) tests were used to determine if there are significant differences (*p* < 0.05) between each product. CVA was applied to TDS results because of its robustness in differentiating samples (Albert et al., 2012; Jager et al., 2014; Pineau et al., 2012).

#### 2.7.3 Analysis of variance

Analysis of variance (ANOVA) was carried out on TDS sensory durations to determine the changes in duration of dominance of sensory attributes of individual product with different intakes. Using the duration of each recorded sensory attribute as explanatory variable, multiple intake TDS data was analyzed according to the following mixed model:

$$\begin{array}{ccc} \mathrm{Duration} & = & \mathrm{Panelists}+\mathrm{Product}+\mathrm{Intake}+\mathrm{Panelists}\\ & & \times \;\mathrm{Product}+\mathrm{Panelists}\times \mathrm{Intake}+\mathrm{Product}\\ & & \times \;\mathrm{Intake} \end{array}$$

where, duration is the time (in seconds) of each recorded attribute, panelist is a random effect, and product and intake were set as fixed factors. Tukey's multiple comparison tests were applied when ANOVA results reached statistical significance to determine if significant differences (*p* < 0.05) exist between means.

#### 2.7.4 Liking while dominant

The present research also used the technique of centering the individual liking while dominant (LWD) values towards the average liking scores given by corresponding panelists to each product. This can ascertain if a dominant attribute leads to increased or decreased liking (Thomas et al., 2015). LWD is the average of liking scores given by the panelists to a product, while the certain attribute was dominant. This average is then computed over selections of the same attribute and is weighted by its duration over the three intakes. All of the individual LWDs were then centered toward the average liking scores specified by the corresponding panelist to all the products (CLWD) (Thomas et al., 2016). Nullity of all these CLWD averages was analyzed by *t*‐test in which number of degrees of freedom is equal to the number of panelists quoting a particular attribute for a particular product minus 1. A CLWD score significantly (*p* < 0.05 or 0.01 or 0.001) > 0 signifies a sensory attribute that stimulates a positive trend of liking when it is dominant. On the other hand, a CLWD score significantly (*p* < 0.05 or 0.01 or 0.001) lower than 0 signifies a sensory attribute that stimulates a negative trend of liking.

#### 2.7.5 Dynamic liking of each intake, satiety, and postconsumption ratings

A mixed ANOVA was performed on the results for overall liking, satiety, perception of healthiness, purchase intent, and satisfaction). Tukey's multiple comparison tests were carried out for the ANOVA results that reached statistical significance (*p *< 0.05).

## 3 RESULTS

### 3.1 Temporal dominance of sensations

#### 3.1.1 Multiple intake TDS curves

**FIGURE 2:**
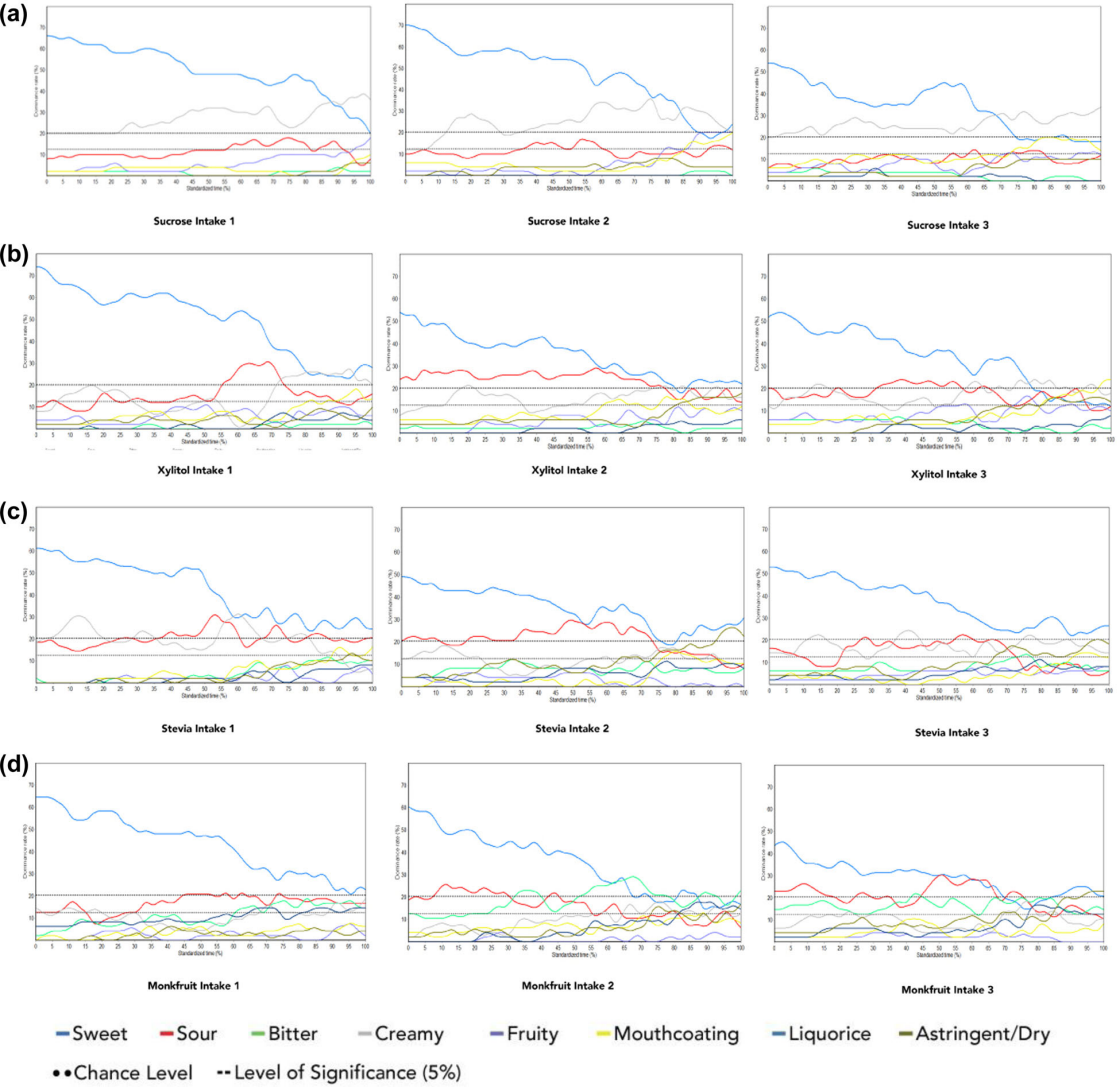
Panel dominance rates (%) of the eight sensory attributes presented in the TDS sessions expressed in standardized time (%). TDS curves are for the different sugars (within column—from top to bottom: sucrose, xylitol, stevia, and monk fruit) over three different intakes (within row—from left to right: first, second, and third intakes, respectively)

Figure 2 depicts the spline smoothed multiple‐intake TDS curves for the four different types of yogurts, one sweetened with 9 g of sucrose, and three sweetened individually with stevia, xylitol, and monk fruit at iso‐sweet concentrations to sucrose. The chance and significance levels were calculated to be between 15 and 20% respectively. Attributes below 20% that is, below significance level were not discussed further. *Sweet* was the first dominant attribute that decreased from a maximum dominance rate of 66–20% (0–100% ST), 70–24% (0–87% ST), and 54–22% (0–74% ST) in the first, second, and third intakes, respectively, of yogurt sweetened with sucrose. In the first intake, *creamy* was dominant between 24 and 100% ST with a maximum dominance rate of 40% at 98% ST in the first intake. *Creamy* was dominant from 15–100% ST with a maximum dominance rate of 34% between 57–63% ST and at 74% ST in the second intake. In the third intake, *creamy* was dominant throughout the evaluation period along with a maximum dominance rate of 34% at 100% ST in the third intake.

*Sweet* was the first dominant attribute that decreased from a maximum dominance rate of 74–28% (0–100% ST), 54–22% (0–100% ST), and 54–20% (0–76% ST) in the first, second and third intakes, respectively of yogurt sweetened with xylitol. *Sour* was the next dominant attribute that decreased from a maximum dominance rate of 74–28% (0–100% ST), 54–22% (0–100% ST), and 54–20% (0–76% ST) in the first, second, and third intakes, respectively of yogurt sweetened with xylitol. *Sour* was another dominant attribute in all the three intakes of yogurt sweetened with xylitol. In the first intake, *sour* was dominant from 55–73% ST with a maximum dominance rate of 32% between 68 and 70% ST. The second intake of yogurt sweetened with xylitol had a longer duration of *sour* that is, from 0 to 76% ST, with a maximum dominance rate of 30% between 6 and 7% ST and 57 and 58% ST. In the third intake, *sour* was dominant between 33 and 56% ST with a maximum dominance rate of 24% at different time points (37–41%, 46–47%, and 53–54% ST) of the evaluation period.

In stevia sweetened yogurt, *sweet* was dominant throughout consumption (0–100% ST) that decreased from a maximum dominance rate of 61%, 49%, and 53% at 0% ST in the first, second, and third intakes, respectively. In the first intake, *creamy* was dominant between 2 and 21% ST and 55 and 67% ST, with a maximum dominance rate of 33% between 59 and 61% ST. *Sour* was dominant between 48 and 61% ST, 71 and 74% ST, and 82 and 85% ST with the maximum dominance rate of 33% at 54% ST. In the second intake, *sour* was dominant between 35 and 72% ST, reaching a maximum dominance rate of 31% at 50% ST. Towards the end of consumption, *astringent* became dominant between 93 and 100% ST, with a maximum dominance rate of 27% at 96% ST. As for the third intake, creamy was dominant between 11 to 16% ST and 37 to 45% ST, reaching a maximum dominance rate of 25% at 42% ST.

During the first intake of yogurt sweetened with monk fruit, *sweet* was the only dominant attribute, with a maximum dominance rate of 65% at the start of evaluation (0% ST), which subsequently decreased down to 23% by the end of evaluation (100% ST). In the second intake, *sweet* was dominant with a high dominance rate of 60% at 0% ST that then decreased until 67% ST. *Sour* was dominant between 8 and 30% ST with a maximum dominance rate of 27% at 12% ST, and *bitter* was dominant between 56 and 75% ST with a maximum dominance rate of 29% at 70% ST. In the third intake, *sweet* had the highest dominance rate of 44% at 0% ST that then decreased until 70% ST. *Sour* was dominant between 0 and 18%, 44 and 67%, and 71 and 76% ST, with a maximum dominance rate of 31% at 52% ST. *Astringent* was only dominant between 93 and 100% ST, reaching a maximum dominance rate of 23% between 95 and 100% ST.

#### 3.1.2 ANOVA of standardized duration of dominant attributes

Table 2 summarises the ANOVA and MANOVA results obtained with the standardized dataset for duration of dominance of each attribute (recorded as percentage of total duration) for yogurts formulated with different sweeteners over three intakes. *Sweet* had a significant main effect at both product (*F* = 5.101, *p* < 0.01) and intake (*F* = 16.11, *p* < 0.0001) levels. *Sweet* was significantly the highest in yogurt sweetened with sucrose compared to yogurts sweetened with stevia and monk fruit. In addition, the dominance duration of *sweet* was significantly the highest for the first intake compared to second and third intakes for all the four products. *Creamy* was significantly the highest in yogurt sweetened with sucrose followed by xylitol, stevia, and monk fruit. *Fruity* was significantly higher in sucrose and xylitol sweetened yogurt. *Sour* was significantly higher in all yogurts sweetened with xylitol, stevia and monk fruit. Stevia‐containing yogurt was significantly the highest in astringency compared to the other three sugars. Yogurt sweetened with monk fruit was significantly higher in *bitter* and *licorice* attributes compared to the other three sugars. In addition, *bitter* and *astringent* were both significantly higher in the second and third intakes compared to the first intake for all the four products.

**TABLE 2:** Analysis of variance (ANOVA) and multivariate analysis of variance (MANOVA) results for duration of dominance of each attribute (in standardised time) for the main effects of products, intake, and their interaction. Mean values per each attribute for each product/intake are provided

	Sucrose	Xylitol	Stevia	Monk fruit	*F*_(Product)_	Intake 1	Intake 2	Intake 3	*F*_(Intake)_		*F*_(Product* Intake)_
**Sweet**	44.380 a	39.813 ab	37.660 b	34.500 b	5.101**	45.990 a	37.880 b	33.395 b	16.11****		1.614
**Sour**	11.033 b	19.367 a	18.240 a	17.187 a	5.029**	15.995 a	17.915 a	15.460 a	0.806		1.263
**Bitter**	1.533 c	2.500 c	6.467 b	14.780 a	44.10****	4.285 b	6.990 a	7.685 a	5.218**		1.397
**Creamy**	26.473 a	16.260 b	16.120 b	9.887 c	15.12****	18.205 a	15.700 a	17.650 a	0.740		0.403
**Fruity**	6.687 a	6.760 a	3.273 b	1.760 b	9.570****	4.045 a	3.815 a	6.000 a	2.928		0.302
**Mouthcoating** **^a^**	7.047 ab	8.160 a	4.747 b	4.973 b	2.777*	4.580 a	6.675 a	7.440 a	2.980		1.926
**Licorice**	0.593 c	1.927 bc	4.387 b	7.607 a	19.53****	3.540 a	3.360 a	3.985 a	0.284		1.809
**Astringent**	3.233 b	6.200 ab	8.073 a	6.253 ab	3.691*	2.840 b	7.130 a	7.850 a	8.995***		0.665
	**MANOVA *F*_(Product)_**				13.69***	**MANOVA *F*_(Intake)_**			3.507***	MANOVA *F*_(Product*intake)_	**0.486**

Significance levels: *5%, **1%, ***0.1%, ****0.01%. Different letters identify significant differences between product groups (within row) according to Tukey HSD. Significant *F*‐values have been highlighted in bold.

^a^ Fisher LSD was used as a post hoc analysis for attribute *mouthcoating* because Tukey's analysis was unable to identify any significant difference between the products even when *F*_(Product)_ was identified to be significant (*p* = 0.042).

Table 3 shows the ANOVA and MANOVA results for duration of dominance of each attribute (recorded as percentage of total duration) for each yogurt product with each intake. Duration of *sweet* was significantly different for yogurts sweetened with sucrose (*F* = 3.788, *p* < 0.05), xylitol (*F* = 6.579, *p* < 0.01), and monk fruit (*F* = 6.362, *p* < 0.01). In addition, duration dominance of *sweet* was significantly higher in the first intake as compared to third intake for yogurts sweetened with sucrose, xylitol, and monk fruit. There was a significant increase in the dominance duration of attribute *mouthcoating* (*F* = 5.541, *p* < 0.01) in the second and third intakes compared to the first intake in yogurt sweetened with sucrose. The dominance durations of *bitter* and *astringent* were significantly higher for the second and third intakes compared to the first intake for yogurt sweetened with monk fruit.

**TABLE 3:** Analysis of variance (ANOVA) and multivariate analysis of variance (MANOVA) results for duration of dominance of each attribute (in standardised time) for each yogurt product within each intake. Mean values per each attribute for each product/intake are provided

Sucrose	Intake 1	Intake 2	Intake 3	*F*_(Intake)_	Xylitol	Intake 1	Intake 2	Intake 3	*F*_(Intake)_
**Sweet**	50.260 a	48.140 ab	34.740 b	3.788*	**Sweet**	50.020 a	35.100 b	34.320 b	6.579**
**Sour**	11.780 a	11.460 a	9.860 a	0.147	**Sour**	16.020 a	23.920 a	18.160 a	1.953
**Bitter**	1.580 a	0.260 a	2.760 a	1.208	**Bitter**	0.860 a	2.320 a	4.320 a	1.552
**Creamy**	27.640 a	25.400 a	26.400 a	0.069	**Creamy**	15.200 a	15.920 a	17.660 a	0.255
**Fruity**	6.000 a	5.840 a	8.220 a	0.533	**Fruity**	6.000 a	5.340 a	8.940 a	1.069
**Mouthcoating**	2.940 b	6.220 ab	11.980 a	5.541**	**Mouthcoating**	6.760 a	9.120 a	8.600 a	0.341
**Licorice**	0.000 a	0.000 a	1.780 a	1.627	**Licorice**	1.900 a	1.780 a	2.100 a	0.068
**Astringent**	0.800 a	3.680 a	5.220 a	2.432	**Astringent**	4.240 a	7.460 a	6.900 a	0.787
**MANOVA F_(Intake)_**				2.494*	**MANOVA F_(Intake)_**				1.010
** Stevia **	Intake 1	Intake 2	Intake 3	F_(Intake)_	** Monk fruit **	Intake 1	Intake 2	Intake 3	F_(Intake)_
**Sweet**	41.680 a	34.740 a	36.560 a	1.434	**Sweet**	42.000 a	33.540 ab	27.960 b	6.362**
**Sour**	20.200 a	20.660 a	13.860 a	1.198	**Sour**	15.980 a	15.620 a	19.960 a	0.956
**Bitter**	4.280 a	7.500 a	7.620 a	0.707	**Bitter**	10.420 b	17.880 a	16.040 ab	3.558*
**Creamy**	19.360 a	11.840 a	17.160 a	1.593	**Creamy**	10.640 a	9.640 a	9.380 a	0.089
**Fruity**	2.100 a	3.020 a	4.700 a	0.868	**Fruity**	2.080 a	1.060 a	2.140 a	0.382
**Mouthcoating**	5.200 a	4.460 a	4.580 a	0.223	**Mouthcoating**	3.420 a	6.900 a	4.600 a	1.224
**Licorice**	2.380 a	5.700 a	5.080 a	1.728	**Licorice**	9.880 a	5.960 a	6.980 a	1.535
**Astringent**	3.780 a	11.020 a	9.420 a	2.997	**Astringent**	2.540 b	6.360 ab	9.860 a	3.348*
**MANOVA F_(Intake)_**				0.920	**MANOVA F_(Intake)_**				1.110

Significance levels: *5%, **1%, ***0.1%. Different letters identify significant differencesv between product groups (within row) according to Tukey HSD. Significant F‐values have been highlighted in bold.

#### 3.1.3 Canonical variate analysis

**FIGURE 3:**
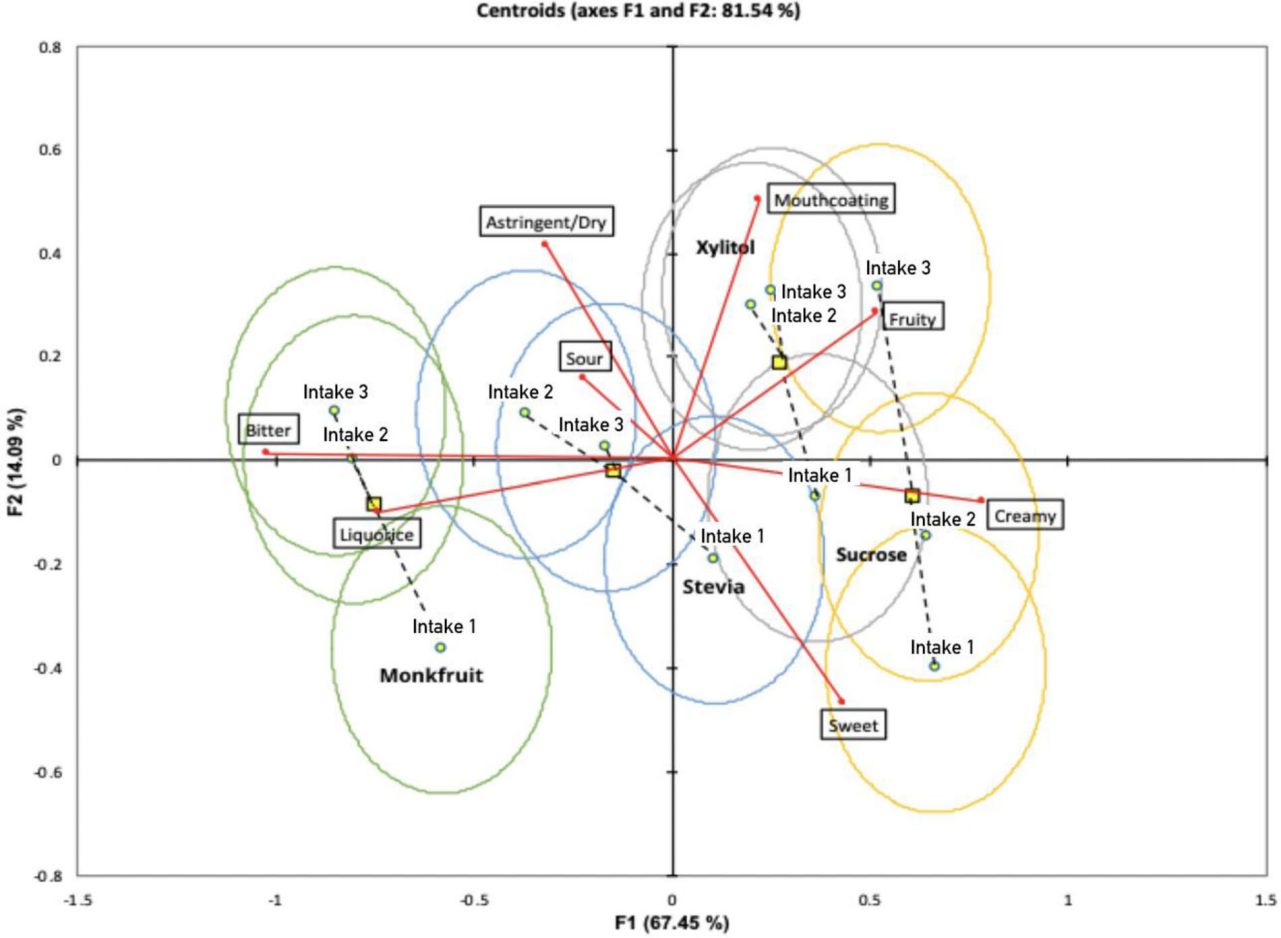
Canonical variate analysis (CVA) of the duration of dominance (in standardised time) for fruit yogurts containing different sweeteners (Green: Monk fruit, Blue: Stevia, Gray: Xylitol, Yellow: Sucrose) over three intakes (*F*_(88,2585)_ = 2.701; *p* < 0.001). Note that 90% confidence ellipses were added to indicate statistical significance. Nonoverlapping ellipses indicate that sample centroid is significantly different, while overlapping ellipses indicate sample centroid are not significantly different

Canonical variate analysis (CVA) is often used to compute product discrimination on the basis of F‐product using the two‐way MANOVA model: Intensity = Product + subject + interactions (Peltier et al., 2015). Hotelling–Lawley MANOVA analysis showed significant differences between the four yogurt samples that varied with sweetener type in terms of dominance duration of sensory attributes as indicated by a significant F‐product value (*F* = 13.69, *p* < 0.001) (Table 2). Moreover, the magnitude of the MANOVA for intake discrimination at a multi‐dimensional level was significant (*F* = 3.507, *p* < 0.001) as shown in Table 2. These differences are better expressed in Figure 3 where all four products with each individual intake are mapped together. Ninety percent ellipses represent the multidimensional confidence interval of the means of dominance duration of sensory attributes. The two canonical variates of CVA factor map accounted for 81.54% of the variation. The first dimension explained 67.45% of the variance and second dimension explained 14.09% of the variance.

The first dimension separated all the four different samples of yogurt according to the type of sweetener used, where positive scores of CVA corresponded to yogurt sweetened with sucrose and xylitol, and negative scores corresponded to yogurt sweetened with stevia and monk fruit. CVA factor map also revealed that yogurt sweetened with sucrose and xylitol were correlated with attributes of *creamy* and *fruity*, whereas yogurt sweetened with stevia and monk fruit were correlated with attributes *licorice* and *bitter*. The second dimension separated the yogurts sweetened with sucrose in terms of intake. The first and second intakes had negative scores along the second dimension, while the third intake had positive scores. This significant difference between three intakes was consistent with the significant MANOVA F‐statistic of yogurt sweetened with sucrose (*F* = 2.494, *p* < 0.05) (Table 3). Moreover, the first and second intakes of yogurt sweetened with sucrose were mainly associated with *sweet*, and the third intake associated with *mouthcoating*. CVA factor maps also revealed that all the three intakes of yogurt sweetened with xylitol, stevia and monk fruit were superimposed over each other indicating no significant difference in terms of duration of dominance of sensory attributes over the three intakes. This is supported by the non‐significant F‐statistic obtained by the Hotelling–Lawley MANOVA analysis (Table 3).

### 3.2 Temporal drivers of liking

Values of centered liking while dominant (CLWD) are summarized in Table 4 to highlight the TDL of yogurts formulated with four different sweeteners over an average of three intakes. *Sweet* was the highest positive TDL in yogurt sweetened with monk fruit as it resulted in an increase of liking by 0.71 (*p* < 0.01) when it was described as dominant by 66% of panelists. Interestingly, liking of yogurt sweetened with xylitol significantly (*p* < 0.05) decreased by 0.45 when *sweet* was described as dominant by 94% of the panelists. In yogurt sweetened with stevia, *sour* significantly (*p* < 0.01) decreased liking by 0.84 when it was described as dominant by 82% of the panelists. *Mouthcoating* was a strong negative TDL in yogurt sweetened with sucrose and significantly (*p* < 0.001) decreased liking by 1.16 when described as dominant by 56% of the panelists.

**TABLE 4:** Temporal drivers of liking

	Sucrose (*n*)		Xylitol (*n*)		Stevia (*n*)		Monk fruit (*n*)	
Attribute	CLWD	% Group	CLWD	% Group	CLWD	% Group	CLWD	% Group
Sweet	0.3	94	−0.45^*^	94	−0.07	82	0.71 ^**^	66
Sour	0.32	60	0.27	68	−0.84^**^	82	0.11	68
Bitter	−0.27	14	−0.45	22	0.46	46	−0.2	50
Creamy	−0.1	98	0.08	82	0.22	84	0.3	68
Fruity	−0.38	54	0.15	58	0.1	36	−0.51	26
Mouthcoating	−1.16 ^***^	56	0.2	68	−0.44	58	−0.4	46
Licorice	−2.48	6	−0.24	22	−0.19	26	−0.01	34
Astringent	−0.01	16	−0.04	40	−0.35	34	−0.69	36

*Note*: The % Group means percentage of panelists (*n* = 50) that cited the specific attribute as being dominant.

Significance levels: *5%, **1%, ***0.1%. Significant centered liking while dominant (CLWD) values have been highlighted in bold.

CLWD value significantly lower than zero indicates a negative driver of liking and CLWD value significantly higher than zero indicates a positive driver of liking.

### 3.3 Effect of sweetener type on dynamic liking, postconsumption attributes and satiety

The associated two‐way ANOVA model for the results of liking showed a significant product effect (*F* = 692.296, *p *< 0.0001), nonsignificant intake effect (*p* = 0.287), and nonsignificant product by intake interaction (*p* = 0.154). Therefore one‐ way ANOVA was used to analyze the results of liking of the yogurts containing different sweeteners over an average of three intakes (Table 5). Yogurt sweetened with sucrose was significantly (*p* < 0.0001) liked the most, followed by yogurt sweetened with xylitol, stevia, and monk fruit (Table 5). Moreover, the four different yogurts were significantly different in terms of healthiness (*F* = 20.28), satisfaction (*F* = 67.86), and purchase intent (*F* = 59.11) at 0.01% level. Yogurt sweetened with sucrose and xylitol were perceived as being significantly the healthiest, followed by stevia and monk fruit sweetened yogurts. Yogurt sweetened with sucrose had significantly the highest satisfaction and purchase intent scores followed by xylitol, stevia, and monk fruit.

**TABLE 5:** Analysis of variance (ANOVA) results showing the effect of sweetener type on the dynamic liking of yogurt and other postconsumption attributes

		Sucrose	Xylitol	Stevia	Monk fruit	*F*_(Product)_
**Dynamic liking**		7.456 a	6.308 b	5.055 c	3.650 d	75.303^****^
**Postconsumption attributes**	Perceived healthiness	5.960 a	5.776 a	4.771 b	3.761 c	20.275^****^
	Satisfaction	7.136 a	5.918 b	4.198 c	2.111 d	67.857^****^
	Purchase intent	6.814 a	5.578 b	3.723 c	1.606 d	59.107^****^

The associated two‐way ANOVA model for the satiety ratings before and after the consumption of yogurt showed significant product effect for hunger (*F* = 10.88, *p* < 0.0001), thirst (*F* = 8.873, *p* < 0.0001), and fullness (*F* = 4.610, p < 0.01), significant condition (pre and post) effect for hunger (*F* = 10.55, *p* < 0.0001), and significant product by condition interaction for thirst (*F* = 2.741, *p* < 0.05). The effects of sweeteners on satiety based on hunger, thirst, and fullness ratings before and after the consumption of all the four different yogurts products are summarised in Table 6. Hunger ratings showed a significant decrease after the consumption of yogurt sweetened with xylitol (*F* = 4.714, *p* < 0.05), stevia (*F* = 14.90, *p* < 0.0001) and monk fruit (*F* = 9.056, *p* < 0.01). Moreover, consumption of yogurt sweetened with monk fruit showed a significant increase in the ratings of thirst (*F* = 8.594, *p* < 0.01).

**TABLE 6:** Analysis of variance (ANOVA) results showing the effects of type of sweetener on the satiety ratings (hunger, thirst, and fullness) before and after the consumption of yogurt

Type of sweetener	Satiety terms	Before consumption of yogurt (Pre)	After consumption of yogurt (Post)	*F*_(Condition)_
**Sucrose**	Hunger	4.206 a	3.964 a	1.055
	Thirst	4.726 a	4.316 a	1.976
	Fullness	4.212 a	4.700 a	2.482
**Xylitol**	Hunger	3.724 a	3.386 b	4.714^*^
	Thirst	3.584 a	3.778 a	0.572
	Fullness	4.882 a	5.088 a	1.129
**Stevia**	Hunger	3.971 a	3.196 b	14.901^****^
	Thirst	4.043 a	3.990 a	0.038
	Fullness	4.382 a	4.555 a	1.118
**Monkfruit**	Hunger	3.257 a	2.792 b	9.056^**^
	Thirst	3.063 a	3.884 b	8.594^**^
	Fullness	4.906 a	5.071 a	0.915

Significance levels: *5%, **1%, ***0.1, ****0.01%. Different letters indicate significant differences between product groups (within row) according to Tukey HSD. Significant *F*‐values have been highlighted in bold.

## 4 DISCUSSION

### 4.1 The dominance duration of sensory attributes varied between intakes in yogurts sweetened with sucrose, xylitol, and monk fruit

Multiple intake TDS methodology allowed the identification of differences in the temporal profile of yogurts formulated with different sweeteners with different intakes. Differences among products in terms of sensory characteristics have been reported to be evident after repeated tasting of products like orange juice sweetened individually with sucrose, sucralose, thaumatin, and stevia (Zorn et al., 2014), chocolate milk formulated with thaumatin and reduced concentrations of sugar (Oliveira et al., 2015), and vanilla milk shake sweetened with sucrose and stevia (Maheeka et al., 2021). It is important to note that in this research yogurt sweetened with sucrose, xylitol, and monk fruit showed significant differences between three different intakes in terms of dominance duration of sensory attributes. These yogurt samples showed a significant decrease in the dominance duration of *sweet* from the first to third intake. This can be attributed to sensory adaptation (Köster, 2003) which can be explained as the reduction in sensitivity, in this case the attribute *sweet* after repeated exposure to it in three different intakes. Lesme et al. (2020) evaluated the texture and flavour perception of fat‐free strawberry yogurts sweetened with sucrose using M‐TDS (Temporal dominance of sensations by modality) combined with multiple intake approach. The authors found that number of spoons had a significant effect on the perception of “sweet” and “sour” attributes that were more dominant for the first spoon compared to third one. A significant increase in the dominance duration of *mouthcoating* with increasing intake in yogurt sweetened with sucrose was also observed. This is in accordance with findings reported by Maheeka et al. (2021) who used the multiple sip TCATA approach. They found that the citation proportion of *mouthcoating* increased from first to the eighth sips for vanilla milk shake formulated using different concentrations of sucrose and a combination of sucrose and stevia. Increase in the dominance of mouthcoating with successive intakes was only significant for yogurt sweetened with sucrose. This was likely due to the significantly different oral processing and hence shear viscosity of this sample as compared to others. Individual differences in the oral mucosa and viscosity of the product has been shown to affect mouthcoating perception (He et al., 2016). Dairy products like ice cream sweetened with xylitol (Khuenpet et al., 2015), chocolate milk sweetened with stevia (Rad et al., 2012), and chocolate milk sweetened with monk fruit (Li et al., 2015) have lower viscosity compared to the same products sweetened with sucrose.

*Bitter* and *astringent* increased in the second and third intakes, respectively in the yogurt sweetened with monk fruit. Bitter, dry and metallic off‐flavours have been reported in food products such as chocolate milk (Li et al., 2015), protein beverages (Harwood & Drake, 2021; Parker et al., 2018), and yogurt (Ban et al., 2020) that were sweetened with monk fruit. Researchers have suggested the use of monk fruit in blends with other sweeteners. It was found that blends containing 50% monk fruit did not elicit any bitterness above the threshold level as compared to blend containing 25% monk fruit or 100% monk fruit in protein beverages (Harwood & Drake, 2021). The decrease in bitterness and astringency with a blend of stevia/monk fruit and sucrose compared to solely stevia or monk fruit were also observed in chocolate milk (Li et al., 2015), kulfi, an Indian traditional frozen dairy dessert (Giri & Rao, 2014), and ice cream (Alizadeh et al., 2014).

Blending of sweeteners has numerous advantages such as synergism in perceived sweetness (Lawless, 1998), and improvement in the overall sensory profile of the product (Schiffman et al., 2007).

### 4.2 Dominance duration of *sweet* varied in yogurts containing different sweeteners

Dominance duration of *sweet* over time differed significantly among the four different yogurts used in this study although they were formulated with iso‐sweet concentrations. In the present study, determination of iso‐sweet concentration of sweeteners employed the sip and spit method (Miele et al., 2019). However, temporal evaluation of sensory attributes of yogurts involved the use of the multiple intake TDS method (Jager et al., 2014) in the present study. Swallowing the yogurt samples when carrying out the TDS procedure has been shown to contribute to differences in perceived sensory intensity when swallowing the sample rather than sipping and spitting (Running & Hayes, 2017).

Dominance duration of *sweet* was significantly longer for yogurt sweetened with sucrose compared to yogurts sweetened by stevia and monk fruit. This is in agreement with findings by Tan et al. (2019) who reported a rapid onset of sweet taste when consuming 10% (w/v) sucrose solution, which lasted throughout the evaluation period of 60 s. This property is not generally common among other sweeteners, which explains the difficulty of replacing sucrose with other sweeteners without compromising the sensory characteristics of the product (DuBois, 1982).

Interestingly, dominance duration of *sweet* in yogurt sweetened with xylitol was not significantly different to yogurt sweetened with sucrose. Similarly, Tan et al. (2019) found that xylitol (9.85% w/v) and sucrose (10% w/v) solutions were similar in terms of peak sweetness citation, time to peak sweetness, and area under curve for sweetness when evaluated using TCATA. Another study showed that probiotic yogurts sweetened solely with sucrose and xylitol were similar in terms of sweet, consistency, and acid aroma when evaluated by conventional descriptive analysis (da Costa et al., 2020). Sweetness of ice cream (Kalicka et al., 2019), and dadih (Malaysian dairy dessert) (Thani et al., 2016) sweetened individually with sucrose and xylitol were also similar. However, coconut milk ice cream sweetened with xylitol and inulin had significantly lower sweetness intensity compared to sucrose. The authors attributed this to the presence of inulin, which increased the mouthcoating perception and decreased the sweetness of the sample.

The dominance duration of *sweet* in yogurts sweetened with stevia and monk fruit was lower than yogurt sweetened with sucrose. This decrease could be attributed to the increased dominance of other attributes like *bitter, licorice*, and *astringent* with the use of these sweeteners. Previous studies have reported dominance of bitter taste followed by metallic flavor and astringency in stevia sweetened yogurt (Pereira et al., 2021), higher area under curve values for bitterness and astringency in monk fruit sweetened protein beverage (Harwood & Drake, 2021), and dominance of bitter taste in chocolate dairy dessert sweetened with stevia (Morais et al., 2014).

Moreover, the maximum dominance rates of attribute *sweet* in yogurts sweetened with sucrose and xylitol were higher than yogurts sweetened with stevia and monk fruit. Pereira et al. (2021) also reported that the maximum dominance rate of sweet in mango skyr yogurt made with 8% sucrose was higher (23%) compared to 0.09% stevia (11%). Wu et al. (2019) similarly found that the maximum citation percentage of sweet was higher in lemonade made with 70 g/L of sucrose (80%) compared to 0.5 g/L (65%) of stevia using single‐sip TCATA.

### 4.3 Yogurt formulated with xylitol was liked more than stevia and monk fruit sweetened yogurts

The highest overall liking score was achieved in yogurt formulated with sucrose followed by yogurt sweetened with xylitol, stevia, or monk fruit. This is in accordance with Costa et al. (2019) who reported that yogurt sweetened with stevia received lower score for overall impression compared to yogurt sweetened with sucrose and xylitol. Moreover, Carvalho et al. (2018) found that as the concentration of stevia increased, overall acceptance for yogurt decreased. However, as the concentration of sucrose and xylitol increased, overall acceptance of yogurt increased. Another study demonstrated that chocolate milks formulated individually with stevia or monk fruit leaf extract as sweeteners were less liked than milks sweetened with sucrose or blends of sucrose with stevia or monk fruit (Li et al., 2015). In a more recent study, protein beverages formulated solely with stevia or monk fruit received lower scores for overall liking compared to beverages formulated with sucrose or sucralose (Harwood & Drake, 2021). Higher overall liking scores for products sweetened with sucrose compared to stevia or monk fruit were also observed for low sugar apple preserves sweetened with stevia (Pielak et al., 2020), aronia kefir (fermented dairy beverage) sweetened individually with 0.40 g of stevia or 0.80 g of monk fruit (Du & Myracle, 2018), and elderberry kefir sweetened with 0.45 or 0.60 g of stevia (Du & Myracle, 2018).

In terms of temporal drivers of liking (TDL), *mouthcoating* significantly decreased liking of yogurt sweetened with sucrose. Past studies have reported that in food products sweetened with sucrose, attributes like astringency, mouthcoating, bitterness, and metallic are detected at low intensities (Gwak et al., 2012; Kim et al., 2015; Reyes et al., 2017), which can have a negative effect on the liking of the product. Surprisingly, *sweet* was a significant negative driver of liking in yogurt sweetened with xylitol. The decrease in liking of yogurt sweetened with xylitol due to dominance of sweet remains unclear. Further work investigating the effect of flavour interaction of might shed some light on this unexpected outcome.

So*ur* was identified as a negative driver of liking in yogurt sweetened with stevia in this study. This is consistent with results reported by Carvalho et al. (2018) who demonstrated that as concentration of stevia in yogurt increased, the scores for overall acceptance decreased. The authors attributed this decrease in acceptance to acid taste, as evaluated by descriptive analysis. Similarly, Pielak et al. (2020) found that increasing the concentration of stevia in low sugar apple preserves was negatively correlated with an overall degree of liking due to increase in the intensity of negative attributes like sour, bitter, astringent, spicy, and metallic.

### 4.4 Yogurt sweetened with alternative sweeteners can decrease hunger after consumption

This research showed that hunger score decreased after the consumption of yogurt sweetened with xylitol, stevia, and monk fruit. Similarly, Ahmad et al. (2018) reported a significant decrease in hunger after consuming the cookies formulated with stevia than cookies made with sucrose. The authors attributed this to the presence of rebaudioside A; a sweet compound found in stevia, which stimulates the release of satiety‐inducing hormones such as peptide YY (PYY), cholecystokinin (CCK), and glucagon‐like peptide‐1 (GLP‐1). An increase in the release of CCK and PYY was also observed by Meyer‐Gerspach et al. (2021) when 20 volunteers received a preload of 50 g of xylitol in 300 ml tap water at 8 a.m. (after consuming a simple restricted carbohydrate standard dinner before 8 p.m. on the evening before) via a nasogastric tube without any significant effect on the glucose levels and insulin release. This indicated a potential of xylitol to be a satiating alternative for sucrose without additional calories.

It is important to highlight that research studies that experimented with artificial sweeteners did not find an increase in the release of satiety‐inducing hormones (Bryant et al., 2014; Steinert et al., 2011). Steinert et al. (2011) found no significant effect on the secretion of gastrointestinal peptides (i.e., GLP‐1, PYY and ghrelin) when 12 healthy subjects received an intragastric infusion of artificial sweeteners (aspartame, acesulfame K, and sucralose) dissolved in water. In another study, 10 healthy subjects who consumed 45 g glucose and 150 mg aspartame or 45 g glucose and 20 mg saccharin in 250 ml water resulted in no significant effects of both sweeteners on the blood glucose response to oral glucose at any point of time (Bryant et al., 2014). In addition, there was no effect on their perception of hunger and fullness. These studies suggest that natural sweeteners might have a better chance in reducing hunger than artificial sweeteners. A significant increase in the score of thirst after consuming a yogurt sweetened with monk fruit was observed. This could be due to the increase in the dominance duration of *astringent* in the third intake of yogurt sweetened with monk fruit. The increased astringency could have led to panelists experiencing increased thirst.

### 4.5 Type of sweetener used to sweeten yogurt influenced ratings of perceived healthiness, satisfaction, and purchase intent

Yogurt sweetened with sucrose and xylitol scored the highest in terms of perceived healthiness. This finding can be attributed to the higher score received by yogurt sweetened with sucrose and xylitol in terms of overall liking compared to the other sugar alternatives. Thus, if panellists liked a particular yogurt, they instinctively rated it higher for perceived healthiness. Luckow and Delahunty (2004) evaluated the consumer acceptance and perceived healthiness of conventional and probiotic blackcurrant juices by informed tasting. They found that panellists who rated the conventional juice higher for overall impression also rated it higher for perceived healthiness. In addition, panellists who rated probiotic juice higher for overall impression also rated probiotic juice higher for perceived healthiness.

Among the different sugar alternatives used in the present study, yogurt sweetened with xylitol scored the highest in terms of satisfaction and purchase intent. Similarly, Costa et al. (2019) found that probiotic yogurt formulated with xylitol scored higher for overall impression and purchase intention compared to yogurt formulated with stevia A (90% of rebaudioside) or stevia B (20% of rebaudioside) on the 1st and 28th day of storage. Lower scores in terms of satisfaction and purchase intent for yogurt sweetened with stevia and monk fruit as compared to sucrose also seems to be consistent with previous studies. Li et al. (2015) reported that chocolate milk sweetened solely with stevia or monk fruit received the lowest scores for purchase intention as compared to chocolate milk sweetened with sucrose or with blends of sucrose with stevia or monk fruit. Similarly, in a study by Salazar et al. (2018) cookies sweetened with 75 and 100% of the extract of stevia received the lowest purchase intention.

## 5 CONCLUSION

The present study was designed to determine the effect of sweeteners on the temporal sensory profile of yogurt evaluated using multiple‐intake TDS methodology and determination of other post consumption attributes. Xylitol was found to be the most suitable sugar substitute for yogurt because there was no onset of any negative sensory characteristics at any point in any intake. Yogurt sweetened with monk fruit and stevia resulted in additional negative attributes like *bitter, licorice*, and *astringent*. Yogurt containing xylitol had the highest ratings for overall liking and other postconsumption attributes (healthiness, satisfaction, and purchase intent) among all the alternative sweeteners. Interestingly, hunger scores significantly decreased with the consumption of yogurt sweetened with xylitol, stevia, and monk fruit. Further work should be carried out to understand how informed tasting and labelling of sugar substitutes used in yogurt can further influence food perception. This will help communicate the benefits of using alternative sweeteners to meet increasing demand for low calorie sweetened yogurt products.

## AUTHOR CONTRIBUTIONS

Diksha Chadha: Conceptualization; Data curation; Formal analysis; Methodology; Writing – original draft; Writing – review & editing. Nazimah Hamid: Conceptualization; Methodology; Supervision; Writing – original draft; Writing – review & editing. Kevin Kantono: Conceptualization; Formal analysis; Methodology; Writing – review & editing. Manon Marsan: Data curation; Methodology

## CONFLICT OF INTEREST

The authors have no conflict of interest.
